# A Manual of Dietetics

**Published:** 1887-01

**Authors:** 


A NEW AND ORIGINAL WORK—A MANUAL OF DIETETICS, by J. Milner Fothergill, M. D.. Physician to the City of London Hospital for Diseases of the Chest (Victoria Park); Hon. M. D. Rush Medical College, Chicago, Ill.; Foreign Associate Fellow of the College of Physicians, Philadelphia. 8vo., extra., muslin. 255 pages. Price, $2.50. New York; William Wood & Co.

This is, indeed, a *manual*,—a hand hook, for it is as concise as is consistent with a clear dissertation on: 1. Food in general, its object, various methods of preparation, etc., and 2. Food in infancy, adolescence and adult life, giving in detail the proper food for acute disease, and for the various chronic and strumous affections, such as phthisis, albuminuria, diabetes, gout, enteric troubles, heart disease, etc. It is divided into two parts, as above, and consists of thirty-three chapters, in which about all that is known about the proper diet for sick folks can be found. Reader's will recall Dr. Fothergill's previous work on indigestion, biliousness and gout, which was written in this same sprightly style, and had a good sale, containing, as it did, such an amount of interesting practical information. Careful physicians should have this book; they are aware that there is as much in proper feeding as in proper medication—perhaps more.

